# CASE REPORT Spontaneous Forearm Compartment Syndrome in a Boy With Hemophilia A: A Therapeutic Dilemma

**Published:** 2013-03-16

**Authors:** Jennifer Kim, Jonathan Zelken, Justin Michael Sacks

**Affiliations:** The Johns Hopkins Hospital, Department of Plastic and Reconstructive Surgery, Baltimore, MD

## Abstract

**Objective**: We present the case of a 14-year-old Factor VIII-deficient patient with no history of trauma, who developed acute spontaneous compartment syndrome of the volar forearm. We also suggest a treatment strategy. **Methods**: Fasciotomy with hematoma evacuation and ipsilateral carpal tunnel release was performed, and the wound was closed with vascular loops in “Jacob's ladder” fashion. Factor infusions were continued overnight. **Results**: The volar forearm compartment was successfully decompressed, and the patient's coagulopathy was managed with appropriate clotting factors. **Conclusions**: Hemophilic patients warrant special consideration and multispecialty care; with replenished coagulation factors and timely surgical decompression, they can expect satisfactory recovery of muscular and neurological function.

Compartment syndrome is a serious condition that occurs when perfusion pressure falls below tissue pressure within a closed compartment. This can result from extrinsic forces, such as a tight cast, or intrinsic forces such as excessive interstitial fluid pressure.^1^ Left untreated, neurovascular compromise may lead to permanent muscle and nerve damage, limb loss, rhabdomyolysis, and death.^1^ While most reported cases are associated with trauma and large-vessel damage, hemophilic patients are uniquely at risk for compartment syndrome. Hemophiliacs are predisposed to bleeding and can develop intra-articular, muscular, or neural hemorrhage.^2^ Accordingly, these patients may warrant multispecialty care by hematology, orthopedics, and plastic surgery. We report a case of a child with hemophilia A (Factor VIII deficiency) who presented to the emergency department with acute, spontaneous compartment syndrome of the volar forearm.

## CASE REPORT

The patient is a 14-year-old African American boy with a history of severe factor VIII deficiency. He has been previously treated for intra-articular bleeds in his knees, and was maintained with 3 time per week doses of 1250 U recombinant factor VIII. The patient presented to the emergency department after missing 2 such doses. He was in his usual state of health until the evening before admission, when he noted pain and swelling of his left forearm and ankle. The pain initially responded to nonsteroidal anti-inflammatory drugs, but the following morning, the patient woke to severe pain and could not move his fingers of the left forearm. He was taken to the hospital and seen in consultation by hematology, plastic surgery, and orthopedic surgery.

At the time of admission, the patient had a factor VIII activity of 1% (normal range: 50%-200%). Recombinant factor VIII was administered to normalize factor levels. He was alert, but in considerable pain, and denied a history of trauma. Physical examination revealed a tense and swollen left forearm with limited elbow and wrist motion secondary to pain. Pain was diffuse but centered about the volar-radial left forearm and radial hand. Sensation was diminished along the same area, as well as the median and radial nerve distributions of the hand. The left radial pulse was palpable. A diagnosis of compartment syndrome was suspected and the patient was taken to the operating room for emergency fasciotomy.

**Figure 1:**
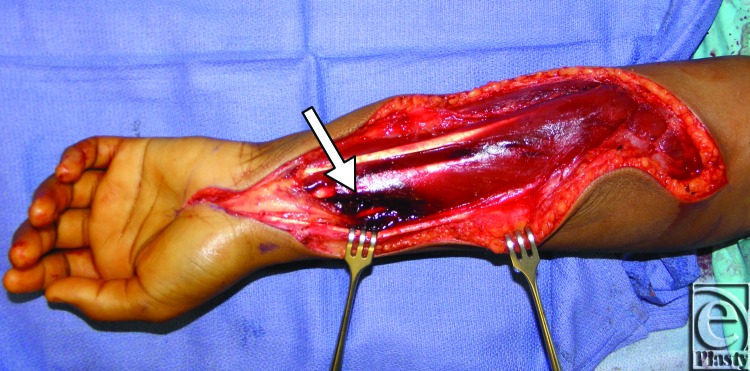
Volar forearm hematoma.

In the operating room, compartment pressures of 72 mm Hg and 10 mm Hg were measured on the left and right volar forearms, respectively. Intraoperative ultrasonography showed diffuse mild edema of the left forearm musculature, with no focal fluid collection or hematoma. Surgical decompression was obtained with generous release of the volar forearm fascia. The compartment was tense; the muscles were swollen and a hematoma of 5 cm^3^ was evacuated from the volar forearm (Fig 1). The fasciotomy preceded ipsilateral carpal tunnel release, after which no tenseness was appreciated in the forearm. An anteroposterior radiograph confirmed that there was no bony involvement, and the wound was closed with vascular loops in “Jacob's ladder” fashion. The arm was placed in a dorsal splint extending from the fingertips to the proximal forearm. Postoperatively, the arm was kept suspended, and factor infusions were continued overnight at 35 U/kg every 6 hours.

## DISCUSSION

Compartment syndrome is a surgical emergency. With volume constraint or increased interstitial fluid production, perfusion pressure may drop below that of the surrounding tissue and result in capillary collapse. Irreversible tissue injury begins around 6 hours after onset, and failure to intervene can lead to necrosis, functional impairment, deformity, renal failure, and death.^1^ Trauma is a common etiology; hemorrhage and edema can elevate compartment pressures above the normal range of 5 to 7 mm Hg.^1^ Onset of the common symptoms—pain, paresthesia, pallor, paralysis, pulselessness, and pressure—in conjunction with trauma, vigorous physical activity, intravenous drug use, envenomation, or anticoagulation therapy should raise concern for compartment syndrome.

Hemophiliacs represent a population uniquely at risk for compartment syndrome. Factor VIII deficiency is the most common congenital coagulopathy. This heritable X-linked disorder is seen in 1 of every 5000 men in the United States.^3^ Clinical symptoms related to intractable bleeding can result from even slight provocation or minor trauma, and can present as large, seemingly spontaneous hematomas and ecchymoses. These patients are at higher risk for iatrogenic bleeding after intramuscular injection, arterial cannulation, venipuncture, and anticoagulation therapy^4^^-^8 and classically present with a history of atraumatic hemarthrosis and intramuscular or intraneural bleeding.

The lack of a clear etiology or conspicuous traumatic injury can make diagnosis a challenge, and descriptions of spontaneous upper extremity compartment syndrome in the context of congenital coagulopathy are scarce in the literature. The first such case report was published in 1906 by Hey-Groves, who detailed the development of Volkmann's contracture in 2 hemophilic patients after minor blunt trauma and subsequent bruising of the forearm.^9^ Seventy-one years later, 4 similar cases were described by Lancourt et al.^10^ In 1981, Madigan et al reported a case of acute compartment syndrome in a factor VIII-resistant patient with hemophilia A, which was successfully treated with activated factor IX and fasciotomy of the anterior compartment and carpal tunnel.^11^ Peripheral nerve lesions resulting from intramuscular hemorrhage have also been described where bleeding into forearm nerve sheaths results in compression of the median or ulnar nerve or both.^12^

Bleeding will typically tamponade before the patient experiences a significant drop in hemoglobin. However, raised compartment pressures can cause significant morbidity in the extremities. Given the short window between symptomatic onset and tissue necrosis, early intervention is crucial and may prove to be not only limb-saving but also life-saving. Hematology should be consulted early to evaluate coagulopathy and monitor factor levels. Infusion of recombinant factor concentrates may be required, after which many patients will experience spontaneous resolution of bleeding.^13^^-^14 If, in such cases, compartment pressures or clinical symptoms remain stable, splinting, rest, and elevation of the extremity may be sufficient to treat the condition. However, once pressures exceed 30 mm Hg and signs of local or systemic deterioration occur, emergent surgical intervention is warranted.^15^

Fasciotomy is the gold standard treatment for compartment syndrome. In patients with hypocoagulable disorders, unique inherent risks must be taken into consideration. Naranja et al^13^ recommend pre- and perioperative factor levels of 50% to 100% to minimize blood loss. Tourniquets are not universally favored in the general population but should be available for additional hemostatic control in these patients. Radiographs must be obtained to rule out bony injury. Ultrasound is useful for evaluating blood flow, collections, and swelling. Hemophilic patients warrant special consideration and multispecialty care; with replenished coagulation factors and timely surgical decompression, they can expect satisfactory recovery of muscular and neurological function.
